# Bioactive Potential of Some *Bacillus thuringiensis* Strains from Macapá, Amazon, Brazil, Against the Housefly *Musca domestica* (Diptera: Muscidae) Under Laboratory Conditions

**DOI:** 10.3390/insects16010027

**Published:** 2024-12-30

**Authors:** Tatiane Aparecida Nascimento, Maria José Paes, Fernando Hercos Valicente, Margareth Maria de Carvalho Queiroz

**Affiliations:** 1 Programa de Pós-Graduação em Biodiversidade e Saúde, Instituto Oswaldo Cruz–Fundação Oswaldo Cruz (IOC/FIOCRUZ), Rio de Janeiro 21040-900, RJ, Brazil; mmcqueiroz@gmail.com; 2 Laboratório Integrado: Simulídeos e Oncocercose & Entomologia Médica e Forense—LSOEMF, Instituto Oswaldo Cruz–Fundação Oswaldo Cruz (IOC/FIOCRUZ), Rio de Janeiro 21040-900, RJ, Brazil; paesmj@yahoo.com; 3 Centro Federal de Educação Tecnológica Celso Suckow da Fonseca, CEFET, Rio de Janeiro 20271-204, RJ, Brazil; 4 Embrapa Milho e Sorgo, Sete Lagoas 35701-970, MG, Brazil; fernando.valicente@embrapa.br

**Keywords:** biological control, bacteria, muscoid flies, pest insects, vector, ultrastructure

## Abstract

The high pathogenic activity of *Bacillus thuringiensis* (*Bt*) strains against various insect orders has positioned it as the most effective, environmentally safe, and sustainable approach to integrated insect management. We aimed to identify *Bt* strains capable of effectively controlling the housefly *Musca domestica*, a species of significant medical, veterinary, and public health concern. Twelve *Bt* strains from Macapá, Brazil, were tested against housefly larvae. PCR was employed to detect genes encoding Cry and Cyt proteins and Vips. Six strains exhibited 70 to 100% larval mortality, with five containing *cry* genes, none harboring *cyt* genes, and four carrying *vip* genes. Scanning electron microscopy revealed the production of crystal inclusions with distinct morphologies: spherical (TR4J, SOL5DM, SOL6RN), cuboidal and bipyramidal (TRO1TN and TRO2MQ), and spherical and bipyramidal (UNI2MA). The potential presence of genes from the *cry1*, *cry2*, *vip1*, and *vip3* families suggests that these strains also exhibit bioinsecticidal activity against other muscoid flies and insect pests across various orders. This study underscores the bioactive potential of these Brazilian *Bt* strains for developing new bioinsecticides.

## Simple Summary

The housefly (Musca domestica) is one of the most common species in urban areas and a mechanical vector of various pathogens affecting human and animal health worldwide. To control this vector, the bacterium *Bacillus thuringiensis* (*Bt*) offers a safe alternative to chemical insecticides that is highly specific, environmentally safe, and economically viable. In this study, we evaluated the effectiveness of 12 *Bt* strains isolated from substrates collected in Macapá, Brazil, on the postembryonic development of housefly. Six strains caused high mortality rates (70–100%) and carried specific *cry* and *vip* genes. Scanning electron microscopy revealed diverse crystal morphologies, suggesting bioinsecticidal potential against this and other pest species. These findings demonstrate the promising potential of Brazilian *Bt* strains for developing bioinsecticides to manage this muscoid dipteran.

## 1. Introduction

*Musca domestica* (Linnaeus, 1758) (Diptera: Muscidae), commonly known as the housefly, is a highly synanthropic muscoid with considerable economic, medical, veterinary, and cosmopolitan importance. It is a recognized mechanical vector for over 60 types of foodborne pathogens and serious diseases, including diarrhea, anthrax, typhoid fever, tuberculosis, and cholera [1,2]. Cases of secondary myiasis caused by *M. domestica* have been documented but remain under-reported in the scientific literature, as noted by Dogra and Mahajan [3] and Salem and Attia [4]. These findings underscore the importance of further research into the bionomics, intrapuparial development, and effective population management of this species [5,6].

Pathogen transmission by *M. domestica* is facilitated by its sucking–licking mouthparts [7]. During feeding, the housefly regurgitates saliva and small droplets from its digestive tract to dissolve the material it consumes while defecating, contaminating food substrates [8].However, pathogen spread is not restricted to regurgitation and defecation [9]. The mouthparts, wings, and leg extremities of this fly also serve as structures for transporting helminth eggs and larvae, further increasing contamination risks [10]. These combined behaviors play a significant role in facilitating disease transmission [11].

The preference of the housefly for diverse substrates—ranging from human and animal waste to decaying plant matter—for its nutritional and reproductive needs plays a critical role in the dissemination of bacterial strains worldwide. This includes species of *Klebsiella* (Enterobacterales; Enterobacteriaceae) which are known to harbor antibiotic-resistant genes [12,13]. Such behavior has significantly contributed to the global proliferation of these pathogens, establishing *M. domestica* as one of the primary pests of medical and veterinary importance [14].

Currently, the control of *M. domestica* predominantly relies on chemical pesticides, many of which fail to adhere to basic biosafety principles. However, the emergence of insecticide resistance and growing environmental concerns about human and animal health have intensified the demand for safer and more sustainable control strategies within the framework of integrated pest and vector management [15,16]. In this context, microbial biological control has emerged as a self-sustaining and effective technique. It has demonstrated efficacy not only in controlling agricultural pests but also in reducing populations of insect vectors, such as flies and mosquitoes [17]. This method leverages pathogenic microorganisms, offering a highly specific and environmentally benign solution. Its advantages include ease of microorganism multiplication, efficient pathogen dissemination, reduced dependence on chemical pesticides, lower rates of insect resistance, and minimized environmental impact and toxicity to human and animal health [18]. Aligned with this approach, the biological agent *Bacillus thuringiensis* (*Bt*) Berliner, 1915 (Caryophanales: Bacillaceae) emerges as a viable alternative for controlling various insect pests and vectors [19].

*Bt* is an aerobic, Gram-positive bacterium that produces crystalline protein inclusions, known as Cry and Cyt proteins or delta (δ) endotoxins, during the stationary phase. Additionally, *Bt* strains synthesize other insecticidal proteins, including vegetative insecticidal proteins (Vips) and secreted insecticidal proteins (Sips) [20,21,22]. Cry proteins are active against lepidopterans, coleopterans, hymenopterans, and dipterans, whereas Cyt proteins exhibit specific toxicity to dipterans in vivo [23]. Despite sharing biochemical properties, Cry and Cyt toxins are neither phylogenetically related nor structurally homologous [24]. These proteins are synthesized during the sporulation phase, with Cyt proteins functioning as cytolytic toxins. Cyt proteins are predominantly produced by *B. thuringiensis* subsp. *israelensis*, as well as by *B. thuringiensis* subsp. *kyushuensis*, *B. thuringiensis* subsp. *jegathesan*, and *B. thuringiensis* subsp. *medellin*, among others [25,26]. Unlike many Cry proteins, Vips target a broader range of insect species, particularly lepidopterans [27]. The nomenclature of Vips differs from that of Cry proteins, as Vips do not form protein crystals [21]. The genes responsible for Vip synthesis are located on high-molecular-weight plasmids, coexisting with *cry* genes [28].

The entomopathogenic activity of *Bt* occurs through the formation of crystals during the sporulation phase, which is driven by protein accumulation. When ingested by specific insects, these crystals release proteins that are activated in their midguts. The activated proteins interact with the epithelial cells, creating pores in the cell membrane. These pores cause disturbances in the insects’ bodies, ultimately leading to their death [29].

Although several *Bt*-based products are available, including VectoBac, VectoLex, VectoPrime, VectoMax, ReMoa Tri, Bactimos (Valent BioSciences Corporation, Libertyville, IL, USA), Teknar (Syngenta, Basel, Switzerland), Aquabac (Becker Microbial Products, Lake Worth, FL, USA), LarvX SG (Meridian Precision Release Technologies, Dalia, Israel), Culinex (Culinex GmbH, Seattle, WA, USA), Bacticide (Biotech International Ltd., Noida, India), Bactivec (Labiofam, La Habana, Cuba), and *Bt*-Horus (Bthek Biotecnologia, Brasília, Brazil), much work remains in regard to addressing a broader range of dipteran vectors, particularly muscoids. These vectors, including species commonly found in urban and rural environments, transmit pathogens of significant public health concern [30,31].

As research advances, it is critical to focus on deploying existing registered and commercialized biopesticides and identifying new *Bt* strains. Particular emphasis should be placed on their molecular characterization rather than relying solely on flagellar antigen H (subspecies) classification [32,33]. This molecular approach is crucial for integrated vector management, as identifying the most effective *Bt* strains for specific vectors, such as *M. domestica*, could significantly reduce dependence on chemical pesticides. Additionally, it plays a key role in insect resistance management and strengthens long-term vector control programs [34,35].

Recognizing the importance of this low-impact system for health applications and harnessing Brazil’s vast biodiversity, this study focused on selecting and characterizing *Bt* strains from Macapá, Amapá State. We assessed their potential for controlling *M. domestica* by investigating their novel insecticidal properties.

## 2. Materials and Methods

This study was conducted at the Integrated Laboratory: Simulids and Oncocercosis & Medical and Forensic Entomology (LSOEMF/IOC), in partnership with the Biological Control Laboratory at EMBRAPA Maize and Sorghum, Sete Lagoas, Minas Gerais, Brazil. We followed the methodology proposed by Merdan [36], with modifications.

### 2.1. M. domestica Colonies

Wild housefly adults were actively collected every two weeks during morning sessions from garbage bins in the Amorim Community (22°52′32″ S; 43°15′02″ W; altitude: 25 m), situated near the entrance to the Oswaldo Cruz Foundation (FIOCRUZ) campus at Rua Sizenando Nabuco, No. 100, Manguinhos, Rio de Janeiro, Brazil. After collection, the flies were transported in an appropriate isothermal box to the LSOEMF/IOC for subsequent identification.

The establishment and maintenance of *M. domestica* colonies followed the procedures described by Nascimento et al. [37].

### 2.2. Bt Strains and Growth Conditions

The *Bt* strains TRO1TN, TRO2MQ, TA5FV, TA1IC, VG1MD, VG2NN, TOR1KC, TOR2VN, UNI2MA, TRAJ, SOL5DM, and SOL6RN were previously collected from various sources in Macapá, including soil samples, organic plant materials, tree trunks, and spider webs (Table 1). These strains, which do not exhibit β-exotoxin pathogenic activity, have been maintained at the EMBRAPA Maize and Sorghum Microorganism Bank and were obtained by the authors for this research. Prior to this study, they were tested exclusively on agricultural pests, specifically *Spodoptera frugiperda* (Smith, 1797) (Lepidoptera: Noctuidae), *Helicoverpa armigera* (Hübner, 1808) (Lepidoptera: Noctuidae), and *Chrysodeixis includens* (Walker, 1858) (Lepidoptera: Noctuidae).

Each strain was cultured on commercial Luria–Bertani (LB) medium supplemented with mineral salts (0.002 g FeSO_4_, 0.02 g ZnSO_4_, 0.02 g MnSO_4_, and 0.3 g MgSO_4_) in Petri dishes. The cultures were incubated at 29 °C for 72 h in a bacteriological incubator to induce sporulation and crystal release. The bacterial biomass was harvested, suspended in autoclaved deionized water in Falcon tubes, and diluted with a 0.05% Tween-20 emulsifier. Spores were then counted using a Neubauer chamber with a phase-contrast optical microscope, Zeiss Axioskop (Oberkochen, Germany).

### 2.3. Selective Bioassays on M. domestica

The experimental design was completely randomized, with four replicates per treatment. Forty neolarvae were placed into 300 mL glass containers (canning jars) containing 80 g of a diet comprising meat meal and wheat bran in a 3:1 ratio. To minimize contamination risk, the diet was exposed to UV radiation in a biological safety cabinet for 20 min prior to use in the bioassays. Subsequently, 4 mL of bacterial solution (10^8^ spore–crystal/mL) from the strains TRO1TN, TRO2MQ, TA5FV, TA1IC, VG1MD, VG2NN, TOR1KC, TOR2VN, UNI2MA, TRAJ, SOL5DM, and SOL6RN was added to each treatment. The control group received 4 mL of autoclaved distilled water. Bioassays were performed in a B.O.D. incubator set to 27 ± 1 °C, with relative humidity being maintained at 70% and a 12 h photoperiod. Mortality rates were assessed 72 h after the initiation of the bioassay, focusing on the postembryonic development of *M. domestica*.

### 2.4. Data Analysis

The data underwent an analysis of variance (ANOVA; *p* ≤ 0.05) followed by factorial analysis using the Scott–Knott test (*p* > 0.05), conducted using the statistical software Sisvar Version 5.6 [38].

### 2.5. DNA Extraction and Molecular Characterization

The *Bt* strains were cultured in an LB medium at 29 °C for 16 h. Subsequently, genomic DNA was extracted using the Wizard^®^ Genomic DNA Purification Kit (Promega Corp., Madison, WI, USA), according to the manufacturer’s instructions. The *cry*, *cyt*, and *vip* genes were detected through PCR reactions using specific primers described in the literature [39,40,41,42,43,44,45] (Table 2).

Each PCR reaction contained 10 ng of DNA, 0.5 µM of each primer, 5 µM of each dNTP, 1× buffer solution, 2 mM MgCl_2_, and 2U of Taq polymerase (KAPA Biosystems, Wilmington, MA, USA) in a total volume of 10 μL. The amplification was performed in a Veriti^®^ 96-Well thermal cycler under the following conditions: initial denaturation at 94 °C for 5 min, 35 cycles of denaturation at 95 °C for one minute, annealing at primer-specific temperatures for one minute, extension at 72 °C for one minute, and final extension at 72 °C for 10 min. The PCR products were analyzed by electrophoresis on a 1% agarose gel using the molecular weight marker 1kb plus (Invitrogen, Carlsbad, CA, USA) for band comparison.

### 2.6. Scanning Electron Microscopy

Scanning electron microscopy (SEM) analysis was performed to examine the morphology and dimensions of crystals found in *Bt* strains that caused the highest average mortality rates in *M. domestica*. The methodology followed the protocols described by Valicente and Souza [46]. The *Bt* strains were cultured on nutrient agar, adjusted to a final pH of 7.5, and incubated at 30 °C for 72 h to ensure complete sporulation. The presence of the spore–crystal complex was then confirmed using a phase-contrast microscope (Zeiss Axioskop, Oberkochen, Germany) at 1000× magnification.

After incubation, the bacterial content was scraped from Petri dishes, and sporulating cells were isolated by centrifugation at 14,000 rpm for 5 min using a specific microtube rotor. The bacterial pellets were then fixed in 2.5% glutaraldehyde in 0.01 M sodium cacodylate buffer for 1 h at room temperature. Subsequently, the samples were washed three times in 0.01 M sodium cacodylate buffer and immersed in 0.5% osmium tetroxide for 1 h. After post-fixation, the samples were dehydrated through a series of ethanol concentrations (25, 50, 75, 90, and 100%), with two immersions at each concentration for 20 min. The dehydrated samples were critical point-dried, mounted on poly-L-lysine-coated coverslips for 48 h, and placed in a tissue culture plate. The samples were then gold sputter-coated and observed using a Jeol JSM-6390LV scanning electron microscope (Garden, UK) on the electron microscopy platform. Micrographs were saved onto CD-RW media for further analysis.

## 3. Results

### 3.1. Bt Virulence

**Figure 1:**
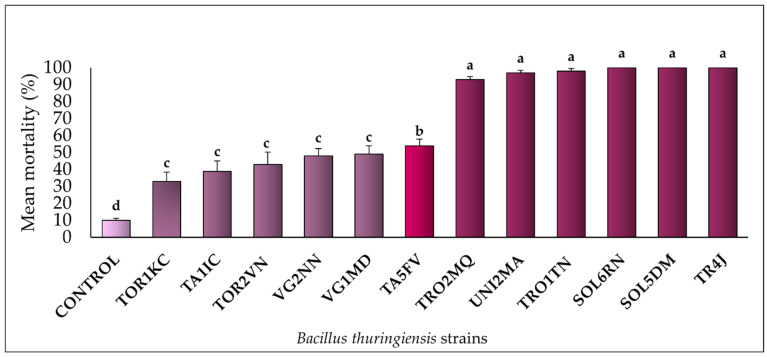
Mortality rates (%) of *Musca domestica* (Diptera: Muscidae) larvae in a selective bioassay with *Bt* strains at a concentration of 10^8^ spores/mL, added to a diet of putrefied ground beef, along with the negative control (autoclaved distilled water). For each treatment, averages marked with the same letter and corresponding to columns of the same color did not differ significantly according to the Scott–Knott test (*p* ≥ 0.05).

**Figure 2:**
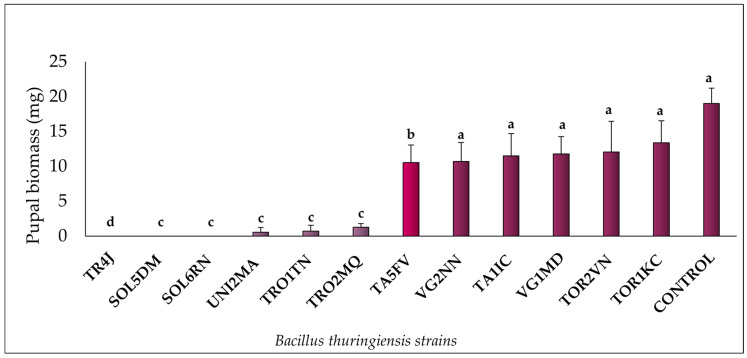
Pupal biomass rates (%) of *Musca domestica* (Diptera: Muscidae) larvae in a selective bioassay with *Bt* strains at a concentration of 10^8^ spores/mL, added to a diet of putrefied ground beef, along with the negative control (autoclaved distilled water). For each treatment, averages marked with the same letter and corresponding to columns of the same color did not differ significantly according to the Scott–Knott test (*p* ≥ 0.05).

The selective bioassay showed that all 12 *Bt* strains tested exhibited pathogenic activity against *M. domestica* larvae. Notably, strains TR4J, SOL5DM, and SOL6RN achieved a mortality rate of 100%, followed by TRO1TN (98%), UNI2MA (97%), and TRO2MQ (93%). The remaining strains displayed mortality rates ranging from 33% to 54%. The control group recorded a mortality rate of 10% (Figure 1). Figure 2 illustrates the average pupal weight values for *M. domestica* across the different experimental groups. Larvae treated with the UNI2MA, TRO1TN, and TROMQ strains exhibited the lowest average pupal weight gains, with values of 0.7 mg, 1.22 mg, and 1.25 mg, respectively. Insects treated with the TA5FV, VG2NN, TA1IC, VG1MD, TOR2VN, and TOR1KC strains also demonstrated a biologically significant reduction in pupal weight, with averages below 14 mg. The control group had an average pupal biomass of 20 mg.

Table 3 shows the significant effect on the duration of postembryonic development of *M. domestica* across different treatments. The larval stage lasted an average of 1.5 to 3.0 days in larvae treated with the 1641, TRO1TN, and UNI2MA strains, indicating a reduction in development time compared to other treatments, where the larval stage averaged six to seven days. A similar trend was observed in the pupal stage, with pupae from larvae fed with *Bt* spore/crystal suspensions of the TRO1TN and UNI2MA strains exhibiting shorter periods than those in other treatments. In contrast, although significant, the deformity rate among emerging adults was below 5%.

**Figure 3:**
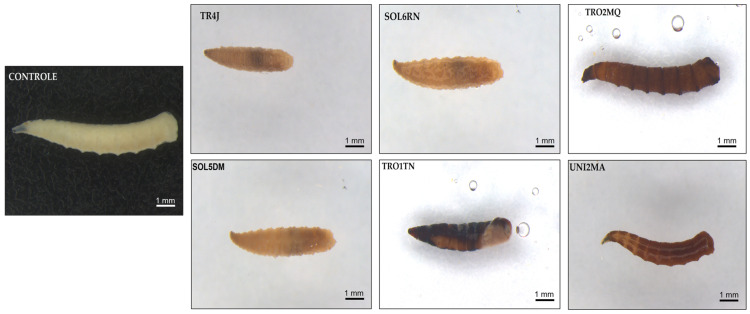
External symptoms of mortality in *Musca domestica* larvae infected by *Bacillus thuringiensis* (10^8^ spores/mL). Control: healthy larvae, with a cream/whitish body color. TR4J, SOL5DM, and SOL6RN: larvae exhibiting partially curved bodies, reduced sizes, darkened abdomens (black coloration), and flaccid body textures. TRO1TN: larva displaying color changes (darkening) over the entire integument, with a necrotic appearance and reduced, curved bodies. TRO2MQ and UNI2MA: larvae showing body darkening (brown and black) and flaccid body textures.

**Figure 4:**
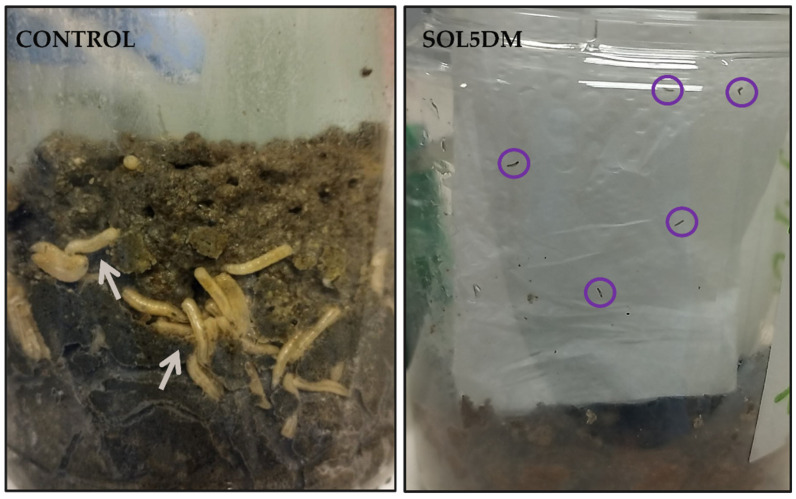
Pathogenicity of *Bacillus thuringiensis* strains in *Musca domestica* larvae. White arrow: *M. domestica* immatures from the control group feeding on the diet. Purple circle: dead *M. domestica* larvae treated with the SOL5DM strain, positioned at the side of the container, indicating abandonment of the diet, with dark-brown coloration on the teguments.

We also observed that surviving larvae, which developed on substrates containing *Bt* spores/crystals (10^8^ spores/mL) from the tested strains, took less time to develop and varied in male-to-female ratio rates. The results show a trend toward reduced average postembryonic development time in specimens fed with meat containing *Bt* spore/crystal suspensions of the TRO1TN and UNI2MA strains. Additionally, macroscopic morphological changes were observed in *M. domestica* larvae treated with *Bt*, contrasting with the control group. These changes included variations in coloration, with a darker tone, and alterations in larval tissue texture, which appeared softer and more flaccid compared to the control group (Figure 3). Furthermore, approximately 12 to 24 h after treatment, most infected larvae ceased feeding and gradually abandoned their diet (Figure 4).

### 3.2. Detection of cry, cyt, and vip Genes

After thoroughly analyzing six *Bt* strains known for their effectiveness against *M. domestica* (SOL5DM, SOL6RN, TRO2MQ TRO1MQ, TRO1TN, TR4J), we found that SOL5DM and SOL6RN amplified *cry2Aa*. TRO2MQ exclusively carried *cry2Ac*, while TRO1MQ and SOL5DM contained *cry2Ad*. Additionally, TRO1TN and TRO1MQ showed the presence of *cry1B*, and TR4J specifically amplified *cry9A*. However, *cry1Aa*, *cry1Ab*, *cry1C*, *cry1G*, *cry1Ea*, *cry9B*, *cyt1*, *cyt2*, and *vip2* were not detected in any of the analyzed strains.

The *vip1* gene was amplified in three strains: TRO1TN, TRO2MQ, and UNI2MA. Conversely, a larger number of strains, including TRO1TN, TRO2MQ, UNI2MA, and TR4J, was amplified for *vip3*. Table 4 summarizes the results, detailing the presence or absence of these amplifications.

Molecular analysis revealed that *vip3* was amplified in 67% of the six strains evaluated, followed by *vip1*, which was detected in 50%. The *cry1B*, *cry2Aa,* and *cry2Ad* genes were amplified in 33% of the strains, while *cry9A* and *cry2Ac* were detected in 17%. Notably, *cry1Aa*, *cry1Ab*, *cry1C*, *cry1G*, *cry1Ea*, *cry9B*, *cyt1*, *cyt2*, and *vip2* were not identified in any of the strains analyzed.

### 3.3. SEM Analysis of Crystalline Inclusions of Bt Strains

**Figure 5:**
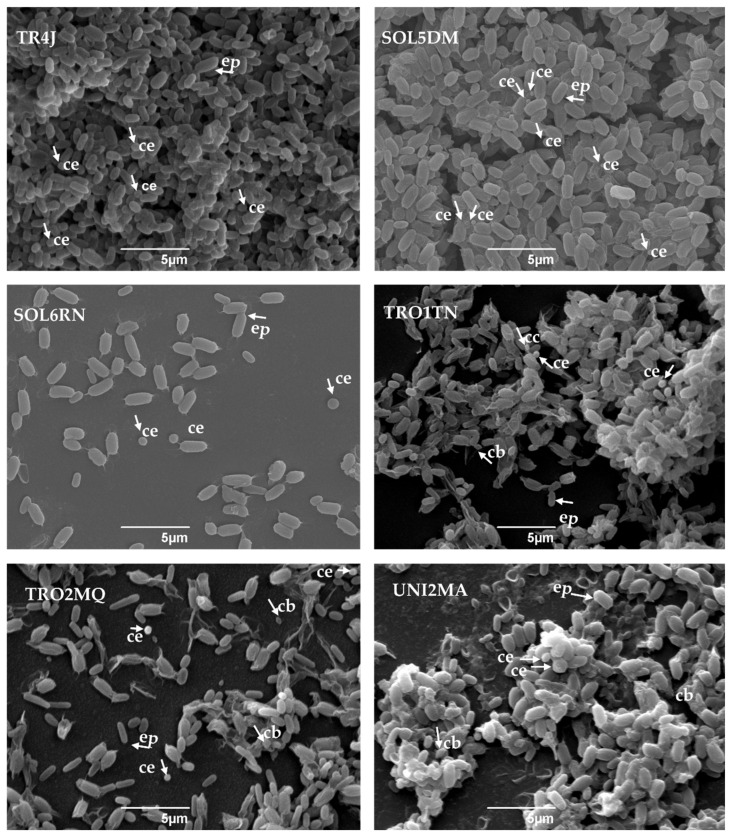
Scanning electron microscopy of the spore–crystal complex of the *Bacillus thuringiensis* strains that showed an average mortality rate of over 70% in *Musca domestica* (Diptera: Muscidae) larvae. ep: spore; cb: bipyramidal crystal; ce: spherical crystal; cc: cuboid crystal (5000× magnification).

SEM analysis revealed that the TR4J, SOL5DM, and SOL6RN strains exclusively produced spherical crystals. In contrast, TRO1TN and TRO2MQ produced a variety of crystal shapes, including spherical, cuboidal, and bipyramidal forms. UNI2MA, on the other hand, exhibited both spherical and bipyramidal crystals. Figure 5 presents the SEM images of crystal morphologies of the different *Bt* strains.

## 4. Discussion

*Bt* is a bacterium commonly found in diverse habitats, including soil, water, litter, grain, deceased or diseased insects, spider webs, and other debris [47,48]. The diversity of *cry*, *cyt*, and *vip* genes in the *Bt* genome may be influenced by the environmental factors present at the collection sites and substrates, as suggested by Valicente and Barreto [49] and Djenane et al. [50]. The strains analyzed in this study were isolated from various substrates, including soil, tree trunks, and spider webs.

The TR4J strain was isolated from a spider web near the Matapí River, while TRO1TN and TRO2MQ were isolated from tree trunks in the Matapí River region. UNI2MA was isolated from soil on the campus of the Federal University of Amapá. Previous studies [50,51,52,53] have shown that analyzing *Bt* strains from diverse ecological and geographic sources results in a weak correlation between their genetic content and the mortality of target insects. This finding highlights the need for further investigation to better understand the broad entomocidal activity of *Bt* strains [54].

The most frequent amplicons were the *vip3* genes, present in four out of six strains (67%), followed by *vip1* in 50% and the *cry1B*, *cry2Aa*, and *cry2Ad* genes, which were amplified in 33% of the strains. These findings are consistent with those of Seifinejad et al. [55], who, in their molecular characterization of 57 strains, identified the presence of *vip* genes in 82.6% of them.

In a survey of insecticidal toxins in 125 *Bt* strains, Espinasse et al. [56] observed the presence of *vip1* and *vip2* in approximately 34.5% of the strains and *vip3* in 52.8%. Similarly, Nascimento et al. [57], in a study focused on the selection and characterization of *Bt* strains against lepidopteran pests, recorded a higher frequency of *vip1* (75%), *vip2* (75%), and *vip3* (67%) in the selected strains.

Considering the higher frequency of *vip* genes in the strains of our study, the efficacy of the *Bt* crystal protein complex may result from the combination of *cry* genes and the insecticidal synergy between Cry proteins and Vips [58]. The coexistence of *vip* with *cry* genes was observed in the TR4J, TRO1TN, and TRO2MQ strains (Table 3), which is consistent with previous studies [59]. The correlation between the occurrence of *cry1*, *cry2*, and *vip* was previously reported by Hernandez et al. [42] during the identification and classification of *cry* and *vip* genes in a collection of 507 *Bt* strains from Spain and Bolivia.

Previous studies, such as those by Wang et al. [60], investigated the interaction between *Bt* toxins Cry9A and Vip3Aa, revealing a strong affinity between these proteins and highlighting their insecticidal synergy, which contributed to high activity against the Asian rice borer. In contrast, Wang et al. [61] found no evidence of synergy between three Cry proteins and Vip3Aa. However, they observed that Vip3Aa had toxic effects on the dipteran *Aedes aegypti* (Linnaeus, 1762) (Diptera: Culicidae), challenging the earlier assumption that Vip3Aa’s insecticidal activity was limited to lepidopteran noctuids.

The high mortality rates observed for *M. domestica* in this study reinforce these findings and highlight the potential of Vips as effective alternatives for controlling pest insects and disease vectors. Moreover, since Vips do not share any sequence or structural homology with Cry proteins, they represent a promising tool for preventing and/or delaying the development of insecticide resistance [62]. Consequently, these findings encourage further investigation into the mechanisms of action of *vip* genes, which remain poorly understood, and emphasize the potential of Vips to expand the action spectrum of *Bt*-based products in controlling economically and health-threatening pests worldwide [63].

Our findings align with those of Uribe et al. [52], who characterized a collection of *Bt* strains from various agricultural and wild ecosystems in Colombia. The authors found that 73% of the analyzed strains reacted with universal primers for the *cry1* genes, highlighting *cry1B* as one of the most common. Similarly, Valicente et al. [43] identified *cry1B* as one of the most frequent genes among the 165 characterized *Bt* strains. The detection of *cry1B* in the TRO1TN and TRO2MQ strains, which demonstrated potential efficacy against *M. domestica*, supports previous evidence that, like other *Bt* proteins, Cry1B is toxic to lepidopterans, dipterans, and coleopterans. It is worth noting that this specific toxicity may be influenced by variations in the insect midgut environment and the interactions between the toxin and its receptors [64].

It is important to note that, while the Cry1 family is generally effective against lepidopteran pests, several of its proteins also exhibit pathogenicity against dipterans [65]. Previous studies [66,67,68] have demonstrated that proteins like Cry1Ab are effective against *Ae. aegypti* larvae; Cry1Ac targets adult *Glossina morsitans* Westwood, 1851 (Diptera: Glossinidae); Cry1Ba shows larvicidal activity against *M. domestica*, *Chrysomya albiceps* (Wiedemann, 1819) (Diptera: Calliphoridae), and *Lucilia cuprina* (Wiedemann, 1830) (Diptera: Calliphoridae); and Cry1Ca is toxic to larvae of various mosquito species. The toxicity bioassays and molecular characterization of *Bt* strains conducted in this study revealed the production of some of these Cry proteins with toxic activity against dipterans. These findings highlight the potential of these strains in controlling muscoids, especially considering the limited availability of effective pesticidal proteins and commercial products for fly management.

The profile of the *cry2A* gene from the *Bt* strains analyzed in this study revealed that *cry2Aa* and *cry2Ad* were the most prevalent genes, occurring in 33% of the analyzed strains, while *cry2Ac* was detected in only one strain (17%). This distribution of *cry2* genes aligns with the findings of Mendoza et al. [69]. They characterized 28 *Bt* strains isolated from the Tijuana–Ensenada region in northwestern Mexico and found a predominance of *cry2Aa* (71%) and *cry2Ac* (14%).

Van Frankenhuyzen [70] and Alzahrani and Crickmore [71] found that the Cry2Aa protein exhibits activity against lepidopterans, dipterans, and hemipterans, while Cry2Ac is effective against lepidopterans and dipterans. In contrast, Cry2Ad is specific to lepidopterans. Considering the action spectrum of Cry2A proteins and their binding site specificity relative to Cry1 family proteins [72], the toxicity assays and *cry2* distribution in the *Bt* strains that we analyzed provide valuable insights into the lethal effects of these proteins on *M. domestica*.

Most PCR reactions yielded the anticipated products, except for those using specific primers targeting *cry1Aa*, *cry1Ab*, *cry1C*, *cry1G*, *cry1Ea*, *cry9B*, *cyt1 cyt2*, and *vip2*, which failed to amplify. This suggests that these genes are not responsible for the toxicity of the strains against *M. domestica*. These findings align with those of Van Frankenhuyzen [70] and Polanczyk et al. [73], which showed that, out of 72 proteins tested against Diptera, only 42 demonstrated activity, indicating that pure proteins encoded by some of these genes did not induce significant mortality in *M. domestica* during bioassays. Additionally, gene frequency variation may be influenced by environmental factors, such as the different locations and substrates from which the strains were collected, which could impact the distribution of *cry*, *cyt*, and *vip* genes [74].

Collections of *Bt* strains encompass a variety of *cry*, *cyt*, and *vip* genes arranged in diverse combinations within their genomes [55,75]. However, it is crucial to emphasize that the mere presence of a specific gene does not necessarily indicate toxic activity [76]. This discrepancy arises because gene presence does ensure bacterial expression [77]; some genes may remain inactive or may be expressed at low levels, reducing their entomopathogenic potential. Nonetheless, identifying the types of genes within the strains is essential for establishing correlations between their genetic profiles and bioinsecticidal efficacy [41].

The pathogenicity of Cry proteins is directly associated with the formation of protein inclusions during *Bt* sporulation, which is triggered by adverse environmental conditions [29,78]. The composition of proteins like Cry and Cyt dictates the morphology of the resulting crystals, demonstrating a strong correlation between crystal structure and protein composition.

For example, bipyramidal crystals are often linked to the presence of Cry1 and/or Cry9 proteins [79,80]. In contrast, cuboidal and spherical inclusions are commonly associated with Cry2 proteins and typically coexist with bipyramidal crystals [81]. These structural characteristics are crucial for the entomopathogenic effectiveness of *Bt* strains, as crystal morphology can influence their interaction with insect gut cells, directly impacting their virulence [82].

Palma et al. [83] and Nair et al. [79] reported that many *Bt* strains feature parasporal crystal proteins that are pivotal to their insecticidal action, with their morphology varying based on the type of the δ-endotoxins and their corresponding genes. In our investigation, only spherical *Bt* crystals were identified in the TR4J, SOL5DM, and SOL6RN strains, all exhibiting 100% mortality against *M. domestica*. This observation aligns with the findings of Loutfi et al. [84], who also identified spherical crystals in *Bt* strains that were effective against dipterans.

When assessing pathogenic activity and characterizing Brazilian *Bt* strains against lepidopteran and dipteran pests, Gitahy et al. [85] and Arsov et al. [86] identified efficient strains that also produced spherical, bipyramidal, and cuboidal crystals along with genes from the *cry1* and *cry2* families that were similar to those observed in the *Bt* strains analyzed in our study. These findings highlight that *Bt* strains with these characteristics exhibit entomopathogenic activity across diverse insect orders, underscoring their potential for developing new biopesticide products.

The housefly’s rapid development, high reproductive capacity, mobility, ability to exploit diverse substrates for reproduction, adaptability to different environments, and propensity to develop resistances to new insecticides pose significant challenges to its control [87,88].

Our findings revealed the genetic diversity among the analyzed *Bt* strains, offering key perspectives on the variability of their entomopathogenic activity and their potential for managing *M. domestica*. Soares-da-Silva et al. [89] also identified Brazilian *Bt* strains pathogenic to *Ae. aegypti*, the vector for several arboviruses. This emphasizes the importance of exploring new strains that may provide novel toxin combinations for the biological control of insect vectors, as demonstrated in our study on houseflies.

The results indicate a trend toward a reduced average time of postembryonic development in housefly specimens that feed on meat containing spore/crystal suspensions of the *Bt* strains TRO1TN and UNI2MA. This reduction may be attributed to the entomopathogenic action of *Bt* on susceptible *M. domestica* larvae. The larvae exhibited symptoms such as loss of appetite and abandonment of their diet, likely due to discomfort or intestinal paralysis. This paralysis may result from the hydrolysis of the crystals in the midgut, which releases active toxins (crystals). These toxins can affect the intestinal epithelium and potentially spread to other body cavities, affecting various organs and systems in the insect.

According to Sebesta et al. [90], the larval stage is always more susceptible to *Bt* than adults, with sublethal doses causing anomalies, deformities, and teratological changes during critical metamorphosis stages. Similar observations were made in the present study, where preliminary laboratory tests suggested that *M. domestica* is susceptible to the tested *Bt* suspensions.

However, further investigations into the external symptoms and histological changes in specimens infected by this pathogen are necessary. A more comprehensive approach will enable a detailed characterization of the effects of these *Bt* strains on *M. domestica* and may help guide more precise and sustainable control strategies for this fly.

Indeed, most screening studies on *Bt* strains prioritize lethal action, focusing primarily on its immediate impact on insects. However, it is equally important to assess sublethal effects, which, although not causing obvious mortality, can significantly influence the dynamics of the target insect [91]. Considering sublethal effects is crucial for integrated pest management programs, as not all field applications result in target-insect mortality. Even products with limited efficacy can affect fundamental biological parameters, such as fecundity, longevity, development rate, and sex ratio [92]. In this context, the present study highlights the variation in the insecticidal potential of bacterial suspensions of this pathogen when applied against *M. domestica.*

Body mass estimation is a crucial parameter in evaluating biological agents as candidates for controlling pests and vector insects. According to Hanski [93], Davidowitz et al. [94], and Chapman et al. [95], the relative success of each insect species largely depends on the larvae’s ability to reach the minimum body weight required for viable pupation. These studies suggest that forming viable pupae with comparatively lower body masses is a strategy for mitigating the adverse effects of competition and/or food restrictions.

Conversely, other researchers, such as Ullyett [96] and Goodbrod and Goff [97], have argued that some muscoids are better adapted to pupate even when their final body mass falls below pre-established standard values compared to other species. Furthermore, both a significant reduction and increase in body mass can be detrimental to an insect’s developmental, sexual, and reproductive performance, depending on the context. In this regard, our study suggests that the sublethal effects of *Bt* exposure led to a reduction in body mass in *M. domestica*—a significant biological change that could considerably impact the species’ viability.

Panizzi and Parra [98] emphasized the importance of examining various aspects during the pupal stage, including the duration of the pupal period, the weight of pupae at a specific age, pupal viability, sex ratio, and the occurrence of body deformities, emphasizing the close correlation between pupal weight and reproductive capacity. They also noted that pupae lose water over time, which justifies the need for weighing them at fixed intervals, such as 24 or 48 h after pupation.

Based on these guidelines, the present study suggests that the ingestion and utilization of the substrate by *M. domestica* larvae exposed to *Bt* spores/crystals were compromised. The variation in the effectiveness of the tested strains can be attributed to a range of factors that are both related and unrelated to the *Bt* mode of action. These factors include the dissolution of the crystal, the activation of the protein, and the binding of the activated protein to receptors in the intestinal epithelium. Consequently, both the amount of food consumed by the larvae and the intestinal dysfunction caused by *Bt* may have interfered with the development time and pupal biomass of the target dipteran.

Miranda et al. [99] evaluated the large-scale larval performance of *M. domestica* using 4000 larvae and 1 kg of different diets based on swine, dairy cattle, and poultry manure, along with a control group fed the Gainesville diet, which consisted of wheat bran, alfalfa meal, and cornmeal. They found that peak larval weight was reached four days after inoculating the larvae with the diet, with no significant differences being seen in the development time to first pupation across the diets. However, the survival rate to pupation varied considerably, with the highest survival rates being seen in the Gainesville (74%), swine (73%), and poultry (67%) diets and the lowest rates being seen in the dairy cattle manure diet (50%). Larval weight also varied between the diets, with larvae fed with swine, dairy cattle, and poultry manure averaging 21 mg, 24 mg, and 25 mg, respectively, while the control group had an average weight of approximately 27 mg.

When comparing these results with those of the present study, which was conducted on a smaller scale (40 larvae fed with 80 g of meat meal and wheat flour per treatment), we noted that the average pupal weight in the control group (19 mg) was adequate for the development of *M. domestica*, maintaining the specimens’ viability throughout the evaluation period. However, it was evident that the larval feeding response after ingesting a diet containing *Bt* negatively impacted their development, leading to a significant reduction in pupal biomass rates for this fly, as shown in Table 2.

Fisher [100] and Norris [101] observed that a stable population typically had a male-to-female ratio of 1:1 or slightly more females than males, which helped ensure the species’ perpetuation. Building on these observations, it is advisable to assess other biological parameters, such as the pre-oviposition period, daily mortality rates for both sexes, and daily egg-laying capacity. These parameters could offer further insights into the impact of *Bt* exposure on the biology and population dynamics of this fly.

Despite the growing interest in prospecting new *Bt* strains, a significant gap remains in the literature regarding their bioactive potential against insect pests and vectors, as well as the characterization of *Bt* strains from the state of Amapá in the Brazilian Amazon, particularly in regard to the content of the *cry* and *vip* genes. In this study, we provide important information on *Bt* strains isolated from samples collected in the city of Macapá, emphasizing the need to perform additional sampling of this species, particularly across different Brazilian states.

Macapá houses four terrestrial ecosystems—forest, cerrado, floodable fields, and mangroves—along with a rich aquatic and estuarine biota. This vast biodiversity offers a unique opportunity for isolating *Bt* strains with promising biological properties [102]. Therefore, exploring these strains is crucial for developing biological pest control strategies, identifying specific entomopathogenic activities, and discovering new approaches to managing local insect pests. The efficacy of the strains TR4J, SOL5DM, SOL6RN, TRO1TN, TRO2MQ, and UNI2MA against *M. domestica* underscores the importance of investigating their potential against other species of flies and pests. Further assessments are needed, as the proteins present in these *Bt* strains may pose risks to insects of medical relevance in both sanitary and veterinary scenarios.

## Figures and Tables

**Table 1:** *Bacillus thuringiensis* strains and their respective substrate samples collected in Macapá, Amapá, Brazilian Amazon.

Strain	Substrate	Region	Coordinates
TRO1TN	Dead tree trunk	Comunidade Areal do Matapí—AP	0°15′17″ N; 51°09′57″ W
TRO2MQ	Dead tree trunk	Comunidade Areal do Matapí—AP	0°15′17″ N; 51°09′57″ W
TA5FV	Spider web inside a tree trunk	Comunidade Areal do Matapí—AP	0°15′18″ N; 51°09′54″ W
TA1IC	Spider web inside a tree trunk	Comunidade Areal do Matapí—AP	0°15′18″ N; 51°09′54″ W
VG1MD	Organic matter (leaves)	Comunidade Areal do Matapí—AP	0°15′18″ N; 51°09′57″ W
VG2NN	Organic matter (leaves)	Comunidade Areal do Matapí—AP	0°15′18″ N; 51°09′57″ W
TOR1KC	Roadside soil	Comunidade Torrão do Matapí—AP	0°14′09″ N; 51°11′03″ W
TOR2VN	Roadside soil	Comunidade Torrão do Matapí—AP	0°14′09″ N; 51°11′03″ W
UNI2MA	Forest remnant	Universidade Federal do Amapá—AP	0°00′26″ S; 51°05′02″ W
TR4J	Spider web near the Matapí River	Comunidade Areal do Matapí—AP	0°15′18″ N; 51°09′56″ W
SOL5DM	Soil close to the Matapí River	Comunidade Areal do Matapí—AP	0°15′29″ N; 51°10′00″ W
SOL6RN	Soil close to the Matapí River	Comunidade Areal do Matapí—AP	0°15′18″ N; 51°09′56″ W

**Table 2:** Primers used for genomic DNA amplification of *Bacillus thuringiensis* strains.

Target Genes	Primers Sequences (5′-3′)	Tm (°C)	Fragment Size (bp)	Reference
*cry1Aa*	TGTAGAAGAGGAAGTCTATCCA	53	272	Cerón et al. [39]
	TATCGGTTTCTGGGAAGTA			
*cry1Ab*	CGCCACAGGACCTCTTAT	55	232	Valicente et al. [40]
	TGCACAACCACCTGACCCA			
*cry1B*	CTTCATCACGATGGAGTAA	55	367	Cerón et al. [41]
	CATAATTTGGTCGTTCTGTT			
*cry1C*	AAAGATCTGGAACACCTTT	58	130	Cerón et al. [41]
	CAAACTCTAAATCCTTTCAC			
*cry1D*	CTGCAGCAAGCTATCCAA	55	290	Cerón et al. [39]
	ATTTGAATTGTCAAGGCCTG			
*cry1Ea*	GGAACCAAGACGAACTATTGC	56	147	Cerón et al. [39]
	GGTTGAATGAACCCTACTCCC			
*cry1G*	ATATGGAGTGAATAGGGCG	55	235	Cerón et al. [39]
	TGAACGGCGATTACATGC			
*cry9A*	CATAATAGGCGATGCAGCAA	53	395	Fagundes [42] *
	CTAACGAGCCACCATTCGTT			
*cry9B*	TCATTGGTATAAGAGTTGGTGATAGAC	60	402	Valicente [43] *
	CCGCTTCCAATAACATCTTTT			
*cry2Aa*	GGGGCGACTAATCTCAATCA	53	318	Fagundes [42] *
	AGGTGTTCCCGAAGGACTTT			
*cry2Ac*	ACAGCAGTCGCTAGCCTTGT	55	475	Fagundes [42] *
	CAAATTGTGGATTGCCGTTA			
*cry2Ad*	ACGATATCGCCACCTTTGTC	53	282	Fagundes [42] *
	AGGTGTTCCTGAAGGGCTTT			
*cyt1*	CCTCAATCAACAGCAAGGGTTATT	52	477	Ibarra et al. [44]
	TGCAAACAGGACATTGTATGTGTAATT			
*cyt2*	ATTACAAATTGCAAATGGTATTCC	50	356	Ibarra et al. [44]
	TTTCAACATCCACAGTAATTTCAAATGC			
*vip1*	TTATTAGATAAACAACAACAAGAATATCAATCTATTMGNTGGATHGG	48	585	Hernández-Rodríguez et al. [45]
	GATCTATATCTCTAGCTGCTTTTTCATAATCTSARTANGGRTC			
*vip2*	GATAAAGAAAAAGCAAAAGAATGGGRNAARRA	48	845	Hernández-Rodríguez et al. [45]
	CCACACCATCTATATACAGTAATATTTTCTGGDATNGG			
*vip3*	TGCCACTGGTATCAARGA	48	1621	Hernández-Rodríguez et al. [45]
	TCCTCCTGTATGATCTACATATGCATTYTTRTTRTT			

* Unpublished data.

**Table 3:** Time (in days) of postembryonic development period of *Musca domestica* (Diptera: Muscidae) larvae fed a substrate based on meat and bone meal and wheat bran, incorporated with *Bacillus thuringiensis* strains at 10^8^ spores/mL, in comparison with the control group under laboratory conditions.

Strains 4 mL *Bacillus thuringiensis* 80 g Diet	Larval Stage (Days) X ± SD	Pupal Stage (Days) X ± DP	Newly Hatched Larvae to Adults (Days) X ± SD	Deformity (%) X ± DP	Sex Ratio X ± DP
SOL5DM	0.0 ± 0.0 c	0.0 ± 0.0 b	0.0 ± 0.0 c	0.0 ± 0.0 c	0.00 ± 0.0 b
SOL6RN	0.0 ± 0.0 c	0.0 ± 0.0 b	0.0 ± 0.0 c	0.0 ± 0.0 c	0.00 ± 0.0 b
TR4J	0.0 ± 0.0 c	0.0 ± 0.0 b	0.0 ± 0.0 c	0.0 ± 0.0 c	0.00 ± 0.0 b
TRO1TN	3.0 ± 0.0 b	4.0 ± 0.0 b	7.0 ± 0.0 c	0.0 ± 0.0 c	0.33 ± 0.4 a
UNI2MA	3.0 ± 0.0 b	5.5 ± 4.6 a	8.5 ± 7.0 b	0.0 ± 0.0 c	0.41 ± 0.5 a
TA5FV	6.0 ± 0.0 a	7.0 ± 0.0 a	13.0 ± 0.0 a	0.0 ± 0.0 c	0.55 ± 0.1 a
VG2NN	6.0 ± 0.0 a	7.0 ± 0.0 a	13.0 ± 0.0 a	0.0 ± 0.0 c	0.48 ± 0.1 a
VG1MD	6.0 ± 0.0 a	7.0 ± 0.0 a	13.0 ± 0.0 a	0.0 ± 0.0 c	0.36 ± 0.0 a
TRO2MQ	6.0 ± 0.0 a	10.0 ± 0.0 a	16.0 ± 0.0 a	0.0 ± 0.0 c	0.64 ± 0.2 a
CONTROL	6.0 ± 0.0 a	7.0 ± 0.0 a	13.0 ± 0.0 a	0.0 ± 0.0 c	0.47 ± 0.0 a
TA1IC	6.0 ± 0.0 a	8.2 ± 5.5 a	14.2 ± 5.5 a	1.0 ± 0.5 b	0.52 ± 0.0 a
TOR1KC	7.0 ± 0.0 a	8.0 ± 0.0 a	15.0 ± 0.0 a	0.0 ± 0.0 c	0.47 ± 0.1 a
TOR2VN	7.0 ± 0.0 a	8.0 ± 0.0 a	15.0 ± 0.0 a	3.0 ± 0.8 a	0.46 ± 0.0 a

Values within a column followed by the same letter are not significantly different at the 5% level according to the Scott–Knott test (*p* ≥ 0.05). Source: the authors.

**Table 4:** Molecular characterization of *Bacillus thuringiensis* strains that are effective against *Musca domestica* (Diptera: Muscidae) for the presence of selected *cry*, *cyt*, and *vip* genes.

*Bt* Strains	Genes															
	*cry*											*cyt*		*vip*		
	*cry1Aa*	*cry1Ab*	*cry1B*	*cry1C*	*cry1G*	*cry1Ea*	*cry9A*	*cry9B*	*cry2Aa*	*cry2Ac*	*cry2Ad*	*cyt1*	*cyt2*	*vip1*	*vip2*	*vip3*
TRO1TN	−	−	+	−	−	−	−	−	−	−	+	−	−	+	−	+
TRO2MQ	−	−	+	−	−	−	−	−	−	+	−	−	−	+	−	+
UNI2MA	−	−	−	−	−	−	−	−	−	−	−	−	−	+	−	+
TR4J	−	−	−	−	−	−	+	−	−	−	−	−	−	−	−	+
SOL5DM	−	−	−	−	−	−	−	−	+	−	+	−	−	−	−	−
SOL6RN	−	−	−	−	−	−	−	−	+	−	−	−	−	−	−	−

(+) presence of the gene; (−) absence of the gene. Source: the authors.

## Data Availability

The data that support the findings of this study are openly available in Mendeley Data at https://data.mendeley.com/datasets/rcyt27txr9/1, accessed on 20 June 2024. DOI: 10.17632/rcyt27txr9.1.
